# 2019 Wilkins–Bernal–Medawar lecture Life begins at 40: the demographic and cultural roots of the midlife crisis

**DOI:** 10.1098/rsnr.2020.0008

**Published:** 2020-03-25

**Authors:** Mark Jackson

**Affiliations:** Wellcome Centre for Cultures and Environments of Health, University of Exeter, Queen's Building, Exeter EX4 4QH, UK

**Keywords:** midlife crisis: history of ageing, Elliott Jaques, American dream, family life

## Abstract

In 1965, the psychoanalyst and social scientist Elliott Jaques introduced a term, the ‘midlife crisis’, that continues to structure Western understandings and experiences of middle age. Following Jaques's work, the midlife crisis became a popular means of describing how—and why—men and women around the age of 40 became disillusioned with work, disenchanted with relationships and detached from family responsibilities. Post-war sociological and psychological studies of middle age regarded the midlife crisis as a manifestation of either biological or psychological change, as a moment in the life course when—perhaps for the first time—people felt themselves to be declining towards death. Although the midlife crisis has often been dismissed as a myth or satirized in novels and films, the concept has persisted not only in stereotypical depictions of rebellion and infidelity at midlife, but also in research that has sought to explain the particular social, physical and emotional challenges of middle age. In the spirit of the pioneering research of John Wilkins, John Bernal and Peter Medawar, each of whom in different ways emphasized the complex interrelations between science and society, I want to argue that the emergence of the midlife crisis—as concept and experience—during the middle decades of the twentieth century was not coincidental. Rather it was the product of historically specific demographic changes and political aspirations—at least in the Western world—to keep alive the American dream of economic progress and material prosperity.

## Introduction

In 1965, the Canadian-born psychoanalyst and social scientist Elliott Jaques introduced a term, the ‘midlife crisis’, that continues to shape Western accounts of ageing, love and loss. Working at the Tavistock Institute of Human Relations in London, Jaques was well known for his studies of organizational structures, introducing terms such as ‘corporate culture’ into contemporary discussions of occupational hierarchies and working practices.^1^ His research was based on empirical studies of institutions such as factories, churches and hospitals. But it was also shaped by his practice as a psychoanalyst and by the theories of Sigmund Freud and Melanie Klein. Psychoanalytical influences are particularly evident in his formulation of the midlife crisis. Jaques had begun to think about the concept in 1952—at the age of 35—when a period of personal reflection was prompted by the conclusion of his own analytical sessions with Klein and by reading Dante's *Inferno*—a poetic account of a midlife journey into, and eventually through, darkness and depression. The ‘beautiful lines’ at the start of the *Inferno*, Jaques wrote many years later, ‘melded with my own inner experiences of the midlife struggle with its vivid sense of the meaning of personal death’.^2^

When Jaques first presented his paper on ‘death and the midlife crisis’ to the British Psychoanalytical Society in 1957, it generated only a muted response and was not accepted by the *International Journal of Psycho-Analysis* until eight years later.^3^ In the published version, which was based on a study of 300 creative artists as well as case histories from his clinical practice, Jaques argued that during the middle years of life, when the ‘first phase of adult life has been lived’, adjustment to a new set of circumstances was necessary: work and family had been established; parents had grown old; and children were ‘at the threshold of adulthood’. The challenge of coping with these pressures, when combined with personal experiences of ageing, triggered awareness of the reality of death: ‘The paradox is that of entering the prime of life, the stage of fulfilment, but at the same time the prime and fulfilment are dated. Death lies beyond.’^4^

According to Jaques, those who reached midlife without having successfully established themselves in terms of marriage and occupation were ‘badly prepared for meeting the demands of middle age’. As a result, they were likely to display what became the clichéd features of a midlife crisis: disillusionment with life; dissatisfaction with work; a desperation to postpone mental and physical decline; detachment from family responsibilities; and infidelity with a younger, more athletic accomplice. It was psychological immaturity, Jaques argued, that generated a depressive crisis around the age of 35 that was energetically masked by a manic determination to thwart advancing years:

The compulsive attempts, in many men and women reaching middle age, to remain young, the hypochondriacal concern over health and appearance, the emergence of sexual promiscuity in order to prove youth and potency, the hollowness and lack of genuine enjoyment of life, and the frequency of religious concern, are familiar patterns. They are attempts at a race against time.^5^

Jacqus warned that, for those who did not work carefully through the psychological anguish of midlife, impulsive strategies intended to protect against the tragedy of death were unlikely to be successful: ‘These defensive fantasies’, he insisted, ‘are just as persecuting, however, as the chaotic and hopeless internal situation they are meant to mitigate.’^6^

As Jaques's turn of phrase became more popular on both sides of the Atlantic, he was regularly cited as the originator of the term.^7^ His concept of the midlife crisis shaped research into the life course and informed self-help and therapeutic approaches to the individual and relational challenges of middle age. Already by the late 1960s, Jaques's work was framing attempts to understand and resolve the ‘search for meaning’ that was thought to typify the midlife identity crisis.^8^ In Britain, the impact of the midlife crisis on marriage inspired efforts to address the personal, familial and social determinants—and consequences—of rising levels of divorce. It also influenced the psychoanalytical approaches to resolving marital tensions adopted by Henry V. Dicks and his colleagues at the Tavistock Clinic.^9^ Elsewhere, the midlife crisis became a notable motif in the work of researchers investigating the impact of life transitions on marriage trajectories, personal identity, and health in men and women—most notably in studies by American authors such as Roger Gould, Gail Sheehy, George Vaillant and Daniel Levinson.^10^ The fantasies of middle-aged men hoping to retain their youthful vigour also figured in literary and cinematic treatments of marriage, love and loss during the middle years—most famously in novels by Sloan Wilson, David Ely, John Updike and David Nobbs.^11^

In the post-war decades, the midlife crisis was understood in two principal ways. On the one hand, problems of midlife were read in terms of the stages and transitions of adult psychological development, an approach that had already been outlined by Granville Stanley Hall, Carl Jung and Erik Erikson earlier in the century.^12^ These writers argued that the inability to integrate various facets of personality and identity across the life course led to depressive crises and attempts to forestall the inevitable march of time. On the other hand, clinicians, self-help authors and marriage guidance counsellors pointed to the impact of biological changes that occurred during middle age: middle-aged spread, baldness, reduced vitality and the hormonal turbulence of the ‘change of life’, not only in relation to menopausal symptoms in women, but also in terms of declining testosterone levels in men during what was referred to as the male climacteric. In this sense, the midlife crisis emerged as just one of a number of conundrums linked to the processes of physiological ageing—what Peter Medawar referred to in his inaugural lecture at University College London in 1951 as a set of unsolved problems in biology.^13^

In this article, I want to historicize understandings and experiences of the midlife crisis in the decades after World War II. In commemoration of the critical studies of John Wilkins, John Bernal and Peter Medawar into the interrelations between science and society, I shall argue that the emergence of the midlife crisis in the post-war decades was not a natural phenomenon linked simply to psychological or biological aspects of ageing. Rather it was shaped by distinctive demographic transitions and socio-cultural disruptions in modern Western societies during the 1950s and 1960s that placed specific pressures on middle-aged men and women and their children. The midlife crisis appeared in response to changes in the duration and stages of the life course, transformations in families and communities, and political aspirations—at least in the Western world—to keep alive the American dream of economic progress and material prosperity.

## Demographic change and the crises of middle age

In 1970, the American developmental psychologist Bernice Neugarten emphasized the manner in which individuals passed through socially, as well as biologically, regulated life cycles: ‘There exists a socially prescribed timetable for the ordering of major life events: a time in the life-span when men and women are expected to marry, a time to raise children, a time to retire.’^14^ This normative pattern, Neugarten continued, was ‘adhered to, more or less consistently, by most persons within a given social group’, providing a yardstick by which peers could measure their own progress through life.

Men and women are aware not only of the social clocks that operate in various areas of their lives but also of their own timing; and they readily describe themselves as ‘early,’ ‘late,’ or ‘on time’ with regard to the major life events.^15^

Recognizing that it was social and cultural contexts—rather than biological age—that served to create norms and expectations regarding age-appropriate behaviour, Neugarten insisted that any attempt to chart the stages of the life cycle should acknowledge the ways in which ‘historical time, life time (or chronological age) and social time’ were all ‘intricately intertwined’.^16^

The standardized life course described by Neugarten was a peculiarly modern, middle-class, Western and—as it transpired—transient phenomenon. It appeared first during the early twentieth century, reached its apotheosis in the decades after World War II, and was already beginning to fragment by the 1980s as career choices and family structures began to change again.^17^ According to the economic historian Michael Anderson, a number of features distinguished the life cycle followed by British populations in the mid-twentieth century: they generally lived longer than previous generations, married and set up home at a younger age, had fewer children, and worked—often in the same job—for a standard period of time set by company and state pension schemes.^18^ This pattern—and its impact on domestic life—was well recognized by contemporaries. In his Reith Lectures, broadcast by the Home Service in 1962, George Carstairs, Professor of Psychological Medicine at the University of Edinburgh, pointed out that women's domestic lives had been transformed by three key social transitions: ‘men and women now tend to marry earlier, to have much smaller families, and complete their families in a much shorter space of time’.^19^ The result, as the British social scientist Richard Titmuss had suggested in 1958, was that women spent a far smaller proportion of their lives concerned with childbearing and maternal care than previous generations: ‘by the time the typical mother of today has virtually completed the cycle of motherhood she still has practically half her total life expectancy to live’.^20^

The impact of this increasingly homogeneous life course was striking, particularly in relation to the experiences and challenges of middle age—a period generally understood to comprise the years between 40 and 60. In the first instance, alterations in the timing of life events and increased life expectancy served to extend the period of adulthood, not only for individuals but also for couples ageing together through starting a family, child-rearing and the flight of children from the parental home to work, further education or marriage. Secondly, smaller family sizes and the tendency for children to be born earlier and within a shorter time frame meant that parents lived and worked longer after their children had been born and left home. By the early 1950s, for example, women were, on average, likely to live for a further 52 years after the birth of their last child, well beyond the menopause and beyond the marriage of their children. Interwar and post-war generations were the first to routinely know—and be able to care for—grandchildren and sometimes great-grandchildren.^21^

Demographic changes and shifts in individual and family schedules also impacted on inheritance, contributing to mounting domestic pressures around midlife. Anderson's analysis shows that those born in 1891 could expect to inherit from their parents at the age of 37, while they still had children at home and when their oldest child was ‘at best only just entering the labour force’.^22^ By contrast, those born in 1946 could expect to wait until 56 before both parents had died, that is well after their children had left home, leaving middle-aged parents in the second half of the twentieth century vulnerable to economic constraints, particularly when they were sandwiched between caring for children and caring for elderly parents.^23^ Significantly, standardization of the life course resulted in a large proportion of the population passing through any of these transitions—from single to married, through parenthood, and on to grandparenthood and retirement—in increasingly short spaces of time.^24^ What had once been phases of gradual evolution, with significant overlap between life stages, became relatively sudden ruptures in life experiences, carrying the potential for personal and domestic crises. By the post-war years, families were more often disrupted by parental separation and divorce than they were by death, as they had been in previous centuries.

According to the historian Howard Chudacoff, the growing homogeneity of experiences through the life course was one factor leading to greater ‘age consciousness’ in American culture.^25^ The construction of age norms during the late nineteenth and early twentieth centuries was encouraged by transformations in the structure of schools and the workplace, by new forms of classification and communication, and by mounting dependency on the clock at home and in the office. Personal awareness of age-related milestones was promoted by a growing consumer economy with its renewed emphasis on the consumption of entertainment, home furnishings, cars, holidays, clothes, and food and drink. In her study of envy in America during the early decades of the twentieth century, Susan Matt has argued that by the 1920s purchasing mass-produced goods was no longer condemned as immoral, but now heralded as a legitimate activity in the pursuit of happiness.^26^ As she points out, middle-class middle-agers who now had sufficient income to indulge in competitive consumption endeavoured to ‘keep up with the Joneses’, a notion that continued to shape British and American behaviour and attitudes long after World War II.^27^

Concerns to match the successes of peers were reinforced by the sales strategies of companies such as Hallmark Cards, which exploited preoccupations with age boundaries by producing birthday cards marking the successful passing of chronological milestones—decades as well as years.^28^ Marketing techniques that played on age as a measure of achievement—or failure—were echoed in advertisements for age-defying tonics and creams that emphasized the need to resist the potential physical, emotional and domestic decay associated with reaching a threshold birthday. From the early twentieth century, it became commonplace not only to measure life in annual or decennial increments, but also to evaluate individual and family progress against age-specific benchmarks of status and success, at home as much as at work. Celebratory evaluations of age and attainment were made possible by greater opportunities for wealth and leisure in the post-war years. But they also created the potential for frustration and self-doubt if women were ‘left on the shelf’, men failed to achieve promotion or couples fell behind their contemporaries in terms of family development and social recognition. Crises of confidence during middle age emerged in response to unfulfilled dreams rendered more visible by the unexpected stresses of work, marriage and family.

Standardization of the life course created a cohort of expectant middle-agers experiencing together greater opportunities for longer, healthier lives during the 1950s and 1960s.^29^ But it also increased the likelihood of people experiencing an assortment of personal disappointments, domestic tensions, occupational discontent and economic pressures as they struggled to keep pace with the demands of an individualistic capitalist society, in which the pursuit of self-fulfilment began to supplant commitments to family cohesion.^30^ It is important to recognize, however, that the demographic stability of the standardized life cycle was not necessarily matched by greater family stability or equality within relationships. Nor were improved life prospects or the capacity to realize personal goals distributed evenly across populations. Rather, opportunities for self-fulfilment across a longer life course varied widely according to economic and cultural circumstances.^31^ In the first instance, it is evident that the modern Western life course—with its advantages and limitations—first appeared among the urban middle classes, who benefited earlier than their rural and working-class peers from reductions in mortality rates and improved standards of living. Although they tended to retain older traditional patterns and rhythms across the life course, which in some ways provided a buffer against rapid social change,^32^ working-class parents and their families aged more rapidly, as George Orwell pointed out in 1941, in an essay on Donald McGill's postcards:

One of the few authentic class-differences, as opposed to class-distinctions, still existing in England is that the working classes age very much earlier. They do not live less long, provided that they survive their childhood, nor do they lose their physical activity earlier, but they do lose very early their youthful appearance. This fact is observable everywhere, but can be most easily verified by watching one of the higher age groups registering for military service; the middle- and upper-class members look, on average, ten years younger than the others.^33^

Orwell's suggestion that the manifestations, and to some extent the meanings, of age were determined primarily by differences in socioeconomic conditions was echoed in mid-century studies that revealed how stages of the life course and experiences of ageing varied according to cultural expectations and aspirations related to family structure and the distribution of financial and caring responsibilities.^34^ In 1967, a comparative study of life-cycle patterns suggested substantial differences in the timing, perception and consequences of major life events in different cultural contexts. Traditional Japanese stem families tended to pass through three stages, defined in turn by: the initial co-location of successive generations; the death of the father and transference of headship to the son; and the subsequent death of the mother with the creation of a new nuclear family. With its emphasis on family values, succession of the family line and support of ageing parents, this structure allowed families to adjust effectively to economic pressures—although it is noticeable that divorce levels were characteristically high in Japan across the twentieth century.^35^ In contrast to experiences in Japan, Chinese joint families, in which all siblings stayed within the parental home, often dissolved because of tensions across and within generations.

In contrast to the social milestones and family structures prevalent in Japan and China, the life course of the American conjugal, nuclear family was defined in terms of the timings of marriage, childbearing, contraction of family size, retirement and old age—critical transitions that sometimes fractured domestic stability.^36^ But Western patterns of family life were not uniform. Steven Mintz and Susan Kellogg's detailed study of American families indicates how adulthood and ageing were not consistent across America, but varied according to race and class. Black American families suffered disproportionately in terms of economic depression, unemployment and early widowhood, altering their experiences and expectations across the life course.^37^ As Mintz has argued elsewhere, variations in family structures or in the rates and timings of marriage and divorce were not necessarily a product of race alone, as has been assumed in some analyses, but of ‘divergent social and economic circumstances’, marked by ‘deepening class differences’ and contrasting occupational and educational opportunities.^38^

Cultural differences in how the life course was constructed, represented and experienced suggest the need to be cautious in accepting uncritically the notion of a standardized life course, or in applying middle-class Western models of middle age indiscriminately to other times and places. While the contours of the life cycle constituted one mode of demographic reality, the boundaries and meanings of life stages were always determined by cultural values and practices. Categories such as adolescence and midlife—and their associated biological and psychological characteristics—‘do not arise in language and practice at random’, as Thomas S. Weiner and Lucinda P. Bernheimer suggested in their study of midlife identity among children of the 1960s.^39^ Rather, they become prominent in particular places at specific times because they capture culturally specific challenges and problems across the life course. Recognition of the cultural specificity of ageing and the ways in which the sequence of life stages are understood in individual, family and social terms also indicates how experiences of the life course are always relational, never entirely personal. Midlife and its crises were shaped by conjugal and intergenerational relationships as well as by personality and circumstances—a set of conditions that explains why not all families or individuals responded in the same way to major life events.

Alternative iconographies of the life course emerged during the twentieth century as the life cycle and its stages were imbued with different symbolic meanings. Ancient, medieval and early modern representations of the life cycle had been dominated by literary and visual images of a journey or pilgrimage through the stages of life, with middle age understood as a period of power and privilege before the predictable waning of physical vigour.^40^ Similar notions of rise and fall certainly populated modern discussions of ageing. In 1921, for example, Granville Stanley Hall referred to the ‘binomial curve’ of life, ‘rising from a baseline at birth and sinking into it at death’.^41^ At the same time, however, the older circle of life, with its cosmological and religious resonance, was being gradually broken by the secular, individualizing imperatives of the Western world, the journey now shadowed by anticipation of its final destination—personal death. Attempts to recast images of decay and death in softer terms failed to dispel the sense of desolation generated by the seeming inevitability of bodily decline and spiritual dissolution during later life. References to the seasons of life, or to safe passages between them, did not eradicate fears of crisis, particularly through the second half of the life course. Mature middle age—depicted as the evening or autumn of life—continued to be regarded as a phase marked by emotional turbulence and fractured identity.

Historical studies of the life cycle—and particularly those that focus on middle age—are limited. Recently, however, scholars have challenged reductive stereotypes of the life course and recognized the complexity of experiences across space and time.^42^ This process has demanded analysis of new sources, most notably literary and cinematic accounts of midlife, as well as personal accounts of ageing. In 1988, Margaret Morganroth Gullette explored the emergence of a new revisionist genre of American fiction—led by Saul Bellow, Margaret Drabble, Anne Tyler and John Updike—in which narratives of resilience and progress helped to dispel earlier fatalistic accounts of decline during and beyond the middle years. In her study of novels written about—and often at—midlife, Gullette teases out how cultural contexts collided with the authors’ personal lives to make possible optimistic visions of the life course. Her analysis of the first three novels in the Rabbit series by Updike (the fourth in the series was completed after the publication of Gullette's work) is particularly pertinent. The complete cycle of novels, published at approximately ten-year intervals between 1960 and 1990, set out the life of Harry ‘Rabbit’ Angstrom at the ages of 26, 36, 46 and 56. Gullette is surely right to read the third novel as a commentary on the potential for midlife reconciliation with past failings, acknowledgement of present fortunes, and hopes for personal and family stability in the future.^43^ After all, it is in *Rabbit is rich* (1981) that Angstrom articulates the sense of freedom and indulgence made possible by reaching the middle years: ‘Middle age is a wonderful country, all the things you thought would never happen are happening.’^44^ But there is another key feature displayed in successive decades of Angstrom's life: namely that any stage of the life course could be scarred by crises, by subtly shifting tensions between biological changes, individual desires, family demands and social expectations.

Notwithstanding the late twentieth-century works of Margaret Drabble, Marilyn French, Doris Lessing, Simone de Beauvoir and others, who exposed the obstacles navigated by women through their middle years, midlife novels exploring the new landscapes of adulthood were largely written by men and were dominated by narcissistic male protagonists who feared waning virility and death.^45^ Scholarly studies carried out during the post-war years—including Elliott Jacques's reflections on the midlife crisis—similarly tended to regard the male life course as the human norm, a factor that framed accounts of the midlife crisis as a masculine phenomenon: Daniel Levinson's study of the ‘seasons of a man's life’ appeared 17 years before his comparable study of women.^46^ Yet, the aspirations and experiences of ageing men should not be taken as representative—or be allowed to reduce the value—of women's experiences across the life course, which possessed its own social, as well as biological, periodicity and meaning.^47^ Although the life cycles of men and women across the twentieth century were sculpted by shared sociopolitical contexts, they were simultaneously structured differently by the gendering of work and love. In a cross-cultural study of perceptions of the adult life course, based on field work carried out during the 1980s, Charlotte Ikels and her colleagues suggested that Chinese men and women in Hong Kong staged their life courses in similar ways in terms of youth, midlife and old age, but that women and men interpreted the freedom and responsibilities associated with particular stages differently.

Women are somewhat more likely than men to link the life course to family and child-rearing while men are more likely to talk about work ability, in terms of strength, drive, competitiveness, and achievements, than women. Both sexes see a reduction in pressures in the 40s and 50s. Women focus on the free time gained, while men talk about seeing the results of their work efforts and finally being in a position where one's words and opinions are respected.^48^

If work and love—or more accurately work and family—provided the principal yardsticks by which men and women respectively measured their lives for much of the twentieth century, certain literary forms reveal nuances in how middle age was narrated according to gender. Lynne Segal has demonstrated how fictional narratives of ageing heterosexual men were marked primarily by concerns about declining sexual potency and the ‘increasingly unrealizable’ life of desire, both of which were key motifs in late twentieth-century representations of the male midlife crisis.^49^ In some women's writings, by contrast, ageing was expressed in more positive tones: for post-war feminist writers such as Germaine Greer, Betty Friedan and Gloria Steinem, who were challenging inequalities within the family as well as addressing social and occupational discrimination, the passing of youthful sexual passion allowed women to be ‘free at last’.^50^ Yet, as Segal shows, this dichotomy between male and female perceptions of shifting sexuality across the life course is overly simplistic. Like men, women ageing through midlife regretted their changing appearance and their declining capacity to express love and desire or to ‘compete with the appeal of younger women’.^51^ The midlife crisis was no less real for women than it was for men.

Post-war scholars conceptualized the stages and transitions of the life course, with their attendant stresses, in different ways. According to the influential feminist sociologist Alice Rossi, academic interest in socialization during childhood, adulthood and old age led to longitudinal and cross-sectional studies that sought to explain the processes of change and stability across the life span.^52^ The work of Marjorie Fiske Lowenthal and colleagues on coping strategies across ‘four stages of life’ illustrates Rossi's point. For Lowenthal, all life events could be stressful. But longitudinal cohort studies of middle-class participants indicated that, while early life changes—including job, college, marriage and parenthood—were regarded as voluntary and socially valued, events in middle age or later life, such as children leaving home or retirement, were felt to be enforced and negative.^53^ Studies by sociologists and economists, in contrast, were concerned not with perceptions and experiences of the life stages themselves, but with the extent to which the timing of events across the life cycle was determined by personal choice about family size and work and the impact of those choices on financial security, levels of unemployment and economic stability. Rossi argued that developmental psychologists operated within a different framework again, preferring to situate ‘any specific phase of life in the broad context of the larger life span’.^54^ The early work of the German-born psychologist Charlotte Bühler on understanding the ‘curve of life’ through biographies was followed in the 1960s by more detailed analyses of self-development within the context of a person's history, comprising dates and events that only made sense when ‘assembled and seen together’.^55^ From this perspective, the Western life course—with its potential for both stability and crisis—was shaped consciously or otherwise by the pursuit of certain goals: the satisfaction of need, effective adaptation, creative expansion and a sense of accomplishment that preserved psychological equilibrium.^56^

Debates about the life course and its crises were not solely academic, certainly in relation to midlife. During the middle decades of the century, the middle-aged were becoming a more visible and more powerful proportion of Western populations. John Benson's study of Britain indicates that both the absolute number and the percentage of the population between the ages of 40 and 59 increased steadily across the early and middle decades of the twentieth century.^57^ Post-war commentators pointed to the rapidly rising proportion of the American population that could be regarded as middle-aged. ‘While our total population increased 98 per cent in the last half-century,’ wrote Thomas C. Desmond, chair of the Joint Legislature Committee on Problems of the Aging, in 1956, ‘our middle agers increased by 200 per cent.’^58^ In the context of commitments to social reconstruction and economic recovery during the Cold War, the middle-aged were at the peak of their earning power and played significant roles in the business and political life of the nation.^59^ There were, however, barriers to the capacity of middle-aged men and women to contribute to social stability and economic growth. A feature article in *Time* magazine in 1966, which used Lauren Bacall as the exemplar of graceful and productive ageing through midlife, claimed that the middle-aged were being devalued by a ‘stultifying Youth Cult’ that was thought to be ‘intimately related to the American denial of death’.^60^ Such discussions neatly captured one of the paradoxes of midlife at mid century: while middle age emerged as a luxury or privilege associated with affluence, it also continued to carry cultural assumptions that any worthwhile measure of life had, by midlife, already been lived.

## Happiness beyond midlife

Demographic trends, shifting family timelines and changing social expectations of age-related achievements created multiple stressors for individuals, couples and families at midlife. After World War II, middle-aged men and women struggled to cope with—and sought to escape from—occupational conformity, the demands of conjugal marriage, the responsibility of raising a nuclear family and the pressure of ‘keeping up with the Joneses’. But, in addition to seeking to evade the constraints of family and work, post-war Western populations were seduced by media images of freedom and self-fulfilment, by political promises of material and spiritual prosperity, and by assurances that a renewed sense of self was attainable after the age of 40. Enticements to pursue happiness after midlife were especially evident in the work of self-help authors, who argued that the middle years need not herald physical and mental decline, but that they might offer fresh opportunities for creativity and contentment.

In an interview published in the *Pittsburgh Press* on 10 April 1917, just four days after America had officially entered World War I, Matilda I. Cruice Parsons, the widow of an army officer, implored American women to ‘train for the duties that war time may bring’.^61^ Drawing on her experiences of training pupils in schools and colleges, Parsons had for some years been advocating a form of ‘New Education’ that emphasized the role of physical exercise in developing the brain and balancing the emotions.^62^ In 1917, she argued that preparing for war was analogous to preparing for ‘wifehood and motherhood’ and demanded that attention be paid to women's physical training for military, as well as domestic, responsibilities. Her comments were directed particularly at ‘the adipose woman of forty’ who had neglected to care for herself as she invested her time and energy in raising a family. She claimed that:

It is a paradox of life that we do not begin to live until we begin to die. Death begins at thirty, that is, deterioration of the muscle cells sets in. Most old age is premature, and attention to diet and exercise would enable men and women to live a great deal longer than they do to-day. The best part of a woman's life begins at forty.^63^

Applied initially to women, during the inter-war and post-war years the expression ‘life begins at 40’ was adopted as a catchphrase by self-help authors on both sides of the Atlantic. During those decades, the term was not merely used to encourage individuals to develop their creative potential. It was also promoted as a mechanism for boosting economic recovery by fostering—or exploiting—middle-class middle-aged aspirations for prolonged youth and personal fulfilment.

**Figure 1.**
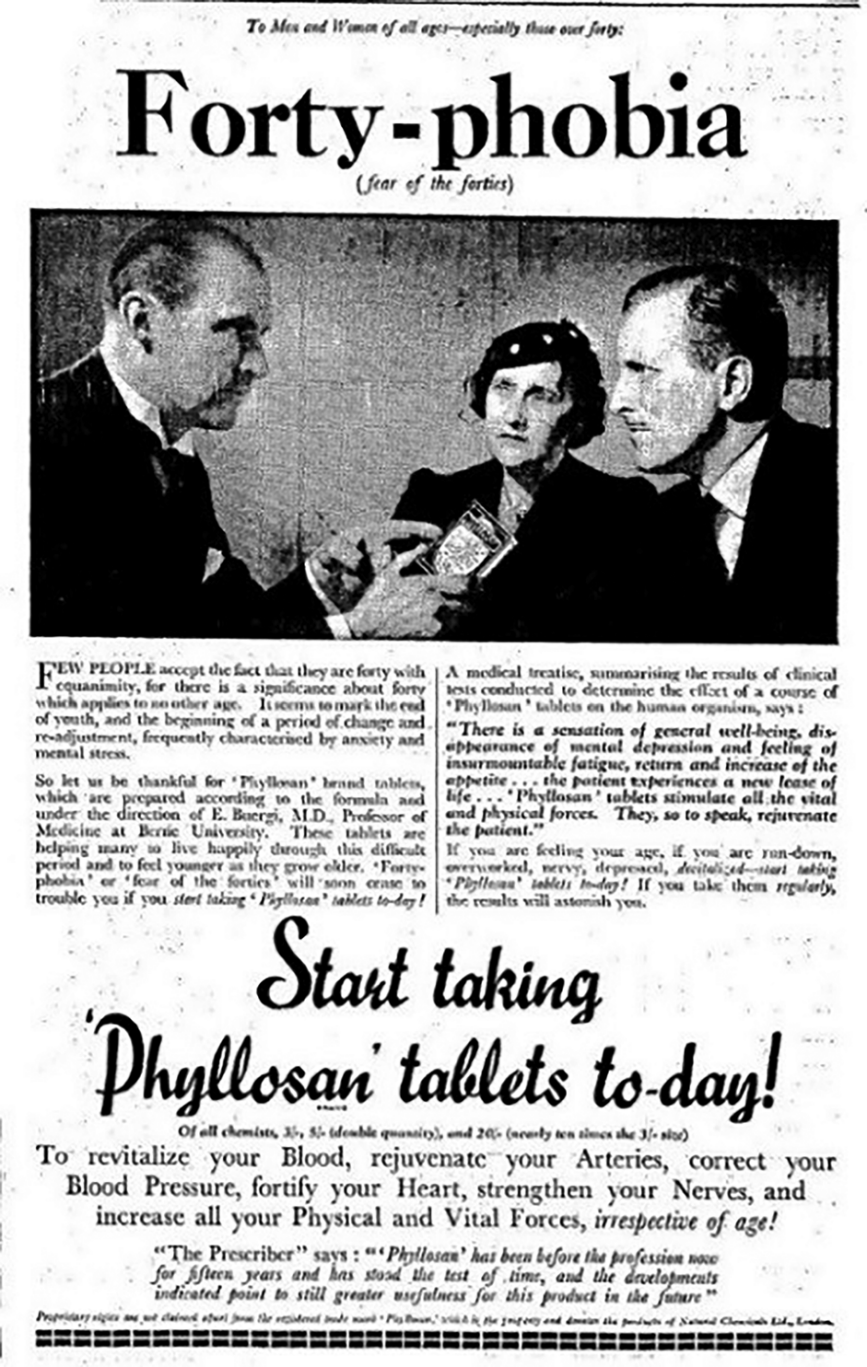
‘Forty-phobia (fear of the forties)’, *Times*, 28 April 1938, p. 19.

Mid-century attempts to forestall age-related changes in mind and body after midlife embraced two principal strategies. On the one hand, they focused on restoring the appearance of youth and vitality or, as Forbes Lindsay had put it in *Harper's Weekly* in 1909, ‘obliterating the footprints of Time’.^64^ Since the early decades of the twentieth century, cosmetics and pharmaceutical companies had marketed soaps, creams, shampoos and hormone preparations designed to restore healthy skin and hair; or they had promoted fitness regimes, supplements, training courses, spa treatments and appliances for arresting middle-aged spread and enhancing attractiveness.^65^ For women in particular, the sales strategies of Elizabeth Arden in America or Boots in Britain testify to popular demand for skin-care products for those over 40.^66^ Commodities for improving appearances and restoring vitality were not limited to the surface of the body, but included tonics designed to steady frayed nerves and combat ‘forty-phobia’.^67^ According to the makers of Phyllosan, 40 marked ‘the end of youth and the beginning of a period of change and re-adjustment, frequently characterised by anxiety and mental stress’. They promised that consuming Phyllosan would allow readers to ‘feel younger as they grow older’ (figure 1).

Men's physical shape and fitness were not ignored in these advertisements for anti-ageing products, particularly given the emerging link between body size, heart disease and the ability to work.^68^ But the majority of marketing strategies and advice literature targeted young and middle-aged women, whose primary value remained tied to their capacity to attract and retain a husband and care for their children. Attention was particularly focused on a woman's face, which provided a ‘canvas upon which she paints a revised, corrected portrait of herself’, as Susan Sontag put it in her caustic critique of the double standard of ageing in 1976.^69^ Such emphasis played on popular beliefs that when husbands started affairs or remarried after divorce, it was usually with far younger women than their wives, who remained largely ineligible for future relationships on account of their age.^70^ Of course, the means to preserve at least the image of youth was not available to all. Rather, it was limited to the middle classes, who had both the desire and the resources—in terms of time and money—to remain relatively young.

The quest for youth could take a second route, one that not merely concealed surface cracks and greying temples, but promised to restore physical, intellectual and economic potency across the life course. In 1932, Walter B. Pitkin, Professor in Journalism at Columbia University and the author of numerous self-help guides between the 1920s and 1940s, used the phrase ‘life begins at forty’ for the title of a popular book that offered readers an antidote to the seemingly inevitable downward spiral of energy and aptitude after midlife.^71^ Enticing his readers with a vision of the excitement, joy and riches on offer in a modern age that had reduced ‘hours of toil’ and increased opportunities for leisure, Pitkin's prescription for self-fulfilment reflected beliefs in the freedom and upward mobility associated with the American dream. American aspirations for health and prosperity had been articulated first in the late nineteenth century, but the principal features of the American dream were most forcefully set out by James Truslow Adams in *The epic of America*, published in 1931.^72^ The American dream, Adams insisted, was

not a dream of motor cars and high wages merely, but a dream of social order in which each man and each woman shall be able to attain to the fullest stature of which they are innately capable, and be recognized by others for what they are, regardless of the fortuitous circumstances of birth or position.^73^

Although the benefits of the dream had been realized more fully in America than elsewhere, Adams acknowledged that growing commitments to ‘business, money-making and material improvement’ had lured Americans into forgetting how to live ‘in the struggle to “make a living”’.^74^ His remedy was to reinvigorate the ‘art of living’. Individuals should acquire and exercise ‘courage, thought and will’ without feeling pressured to conform or to worry about what ‘Mrs Jones’ might be thinking or doing—a sentiment also embodied in Pitkin's prescription for happiness in later life.^75^

After the publication of Pitkin's book, ‘life begins at 40’ became a fashionable slogan in Britain as well as America, providing the basis for the 1935 film comedy *Life begins at forty*.^76^ The phrase was also satirized in popular culture. In the 1942 film *Life begins at eight-thirty*—based on Emlyn Williams's play *The light of heart*—the central character struggles with what was emerging during the middle years of the twentieth century as a familiar pattern of midlife challenges: a failing career, growing debt, addiction to alcohol and the fragmentation of family life.^77^ Intended as comic entertainment, *Life begins at eight-thirty* deftly captured the hopes and disappointments of a generation of middle-agers in a privileged Western world. The term ‘life begins at 40’ also attracted the—largely disparaging—attention of scholars. In their 1956 study on women's dual roles at home and work, the sociologists Alva Myrdal and Viola Klein were sceptical about advertisements that tried to ‘persuade women that they can look 20 at 65’, and dismissed the ‘element of pep-talk’ embedded in claims made by Pitkin and others that it was possible to remain glamorous after 40. However, they did acknowledge that such claims revealed a public desire to maintain active healthier lives and more appealing bodies through and beyond middle age.^78^

Contemporary faith in self-improvement through the forms of materialistic consumption promoted by Pitkin had already been parodied in Lewis Sinclair's novel of 1922, *Babbitt*, in which the principal character's selfish pursuit of prosperity and civic status—and his eagerness to hide his hint of ‘fatness’—threatened to destroy his family before he recognized the emptiness and futility of his grandiosity and ‘crept back’ to his wife.^79^ But, as Myrdal and Klein recognized, the key message in Pitkin's work—that modern populations could now progress from ‘making a living’ to ‘living’—proved attractive to aspiring middle-class middle-agers keen to escape from the rat race of corporate life, generate greater wealth and display their well-earned capacity for leisure.^80^ Pitkin accepted that metabolism and vitality decreased with age and that midlife transitions might be the result of ‘some obscure shift in the endocrine balance’.^81^ He insisted, however, that biological limitations need not prevent people from living more contentedly as they aged. Fulfilment in later life was becoming more attainable in a period when standards of living and opportunities for leisure were becoming matters of national, as well as personal, interest.^82^ Pitkin argued that the increased capacity to afford goods, pursue leisure activities and make the most of their time allowed the middle-aged to attain the ‘emotional poise which underlies enduring happiness’.^83^ ‘Happiness’, he suggested, ‘comes most easily after forty.’^84^

Echoing popular refrains about the passing of years and decades, Pitkin stressed in particular the value of time for readers concerned about approaching old age and death: ‘Time is neither a medium nor is it exchangeable. It is the inmost stuff of life itself.’^85^ But his invocation to spend time, as well as money, wisely across the life course in order to prolong health and happiness was not a disinterested form of advice to beleaguered middle-aged men and women. In addition to promoting Pitkin's own success, *Life begins at forty* was a prescription for social stability and recovery from the Great Depression. He claimed that austerity and automation had adversely affected job opportunities for young people, reducing their capacity to purchase commodities or engage in creative hobbies.^86^ Pitkin's manifesto for personal fulfilment after the age of 40 was underscored by a belief that economic growth, and indeed America's future, now depended primarily on harnessing the spending power, managerial capacities and wisdom of the middle-aged. Only then would it be possible to expand employment prospects for the young, encourage independence and self-sufficiency, promote longer lives in better health, and provide opportunities for leisurely self-realization across the life course.^87^

In his own guide to healthy living, first published in 1965 and aimed at those approaching what he regarded as the new midlife threshold of 50, Pitkin's son reflected on the impact of his father's work. By focusing popular and professional attention on ‘*the subjective, or inner life* of the middle-aged and aging person’, he argued, *Life begins at forty* had encouraged readers to believe that life could be creative, happy and challenging after midlife. In the process, he claimed, his father had launched ‘a whole industry’ of inspirational literature for the middle-aged.^88^ As his son suggested, and as historians have noted, Pitkin senior was by no means the only writer of lifestyle manuals for the middle-aged during the middle decades of the twentieth century.^89^ Numerous books on diet, relaxation and yoga were marketed directly at those over 40 years of age to help them counter the physical and psychological effects of stress, tension, poor diet, limited exercise, excessive work and inadequate rest.^90^

Beliefs that a ‘full and happy life is a balanced one’—particularly for the overworked middle-aged male executive—figured strongly in advice literature for those at midlife.^91^ In their 1938 discussion of the merits of relaxation in a frenzied world where few could ‘attain equilibrium’, the British physician E. J. Boome and the speech therapist M. A. Richardson emphasized the role of relaxation in achieving equanimity: ‘The balance and stability which can be obtained by the practice of relaxation enables a man [*sic*] to face and deal fearlessly with responsibility and trouble instead of trying to evade them.’^92^ Guides to yoga stressed the value of moderation in diet and work, alongside breathing exercises and postures, for those over 40 seeking to retain their youthful energy as well as their figures.^93^ And Gayelord Hauser's best-selling *Look younger, live longer* focused on the manner in which readers could ensure good health in later life not only by adopting balanced diets, but also by balancing their personality, mind, activities, emotions, recreation, friends, budget and marriage.^94^

Arguments for balancing work and leisure in order to improve health across the middle years of life were adopted by researchers in the biomedical and social sciences on both sides of the Atlantic during the middle decades of the twentieth century. Nadina R. Kavinoky was a Swiss-born gynaecologist and the first female president of the American National Council on Family Relations, an organization that had been founded in 1938 to strengthen marriage and family life. In 1944, she claimed that both individual and family mental stability depended on an appropriate balance of work and rest.^95^ Recreation, in particular, facilitated the rehabilitation of ‘men wounded in body and spirit’ and enhanced the capacity of couples and their children to cope with the physical and emotional demands of modern living.^96^ In Britain, too, advice for the middle-aged emphasized the importance of relaxation as a means of counteracting the impact of stressful lifestyles on health.^97^ Yet, there were alternative approaches to healthy ageing that valorized work rather than leisure. According to some writers, both women and men needed to work in order to foster feelings of self-worth: while, for men, satisfaction came from the office or factory, for women their ‘real work was not the monotony of cleaning but the more significant, ennobling job of raising a family’.^98^

Advice literature for both men and women after the age of 40 was coloured by the prejudices of middle-aged men, as well as by social commitments to bolstering marriage and the nuclear family after the war. The importance of maintaining a healthy work–life balance without compromising family stability structured recommendations for women's self-realization in later life, as well as dictating debates about paid employment for women.^99^ According to the older Pitkin, wives and mothers whose children had left home and whose husbands were ‘sunk deeply in the miry ruts of their own business offices’ needed to identify educational and career opportunities that utilized their skills and experience in order to prevent boredom, unhappiness and periods of personal crisis.^100^ By contrast, educated college women needed to temper their commitment to work with more opportunities for play.^101^ In a rather cursory chapter on the travails of ‘idle women of middle age’, Pitkin argued that balancing work and leisure in these ways would enable women in their 40s and 50s to achieve a greater sense of utility and fulfilment in later years.^102^

There were alternatives to Pitkin's heavily patriarchal—and capitalist—tone, at least in some parts of the world. In offering advice to middle-aged women in the 1950s and 1960s, the Canadian gynaecologist Marion Hilliard partially mirrored Pitkin's conservative expectations of women as they aged through—and beyond—marriage and the nuclear family. In particular, she reiterated traditional beliefs in women's principal role in maintaining marital happiness and domestic stability and in the biological determinants of women's physical and emotional wellbeing. At the same time, however, she challenged beliefs that women should invest energy in attempting merely to retain their youthful looks, or in ‘grappling fiercely with such an inevitable force as time itself’, as she put it in 1963.^103^ She insisted that married women should be able to work beyond the home, recognizing that wives, like their husbands, needed the satisfaction of occupation not only to combat the tiredness associated with the demands of motherhood, but also to restore self-esteem. ‘Women need to work to gain confidence in themselves’, she wrote in 1956. ‘Women need to work in order to know achievement. Women need to work to escape loneliness. Women need to work to avoid feeling like demihumans, half woman and half sloth.’^104^

Hilliard was not alone in promoting the benefits of work and recreation for middle-aged women in the post-war years. During the 1960s and 1970s, self-help books were increasingly written for—and by—women in their 40s and 50s. Some authors warned that middle age could bring increased risk of heart disease, cancer and mental illness; or they focused on strategies to mitigate the emotional and physical impact of the menopause. But many writers adopted a more optimistic tone, encouraging women as well as men to capitalize on the freedom and independence that had been made possible by earlier marriages, smaller families and growing affluence—at least among the middle classes. Middle age might not be a period of crisis, but an opportunity for change. If energy was channelled appropriately, midlife could be a transitional moment in the life course, a moment at which men and women could escape from the constraints of corporate and family life and begin—at last—to realize youthful dreams of plenty.

Not everyone agreed that the pursuit of individual happiness in middle age was constructive. In Britain, marriage guidance counsellors blamed rising levels of separation and divorce on narcissism and the atomistic tendencies of modern life.^105^ In the clinics of American psychoanalysts such as Edmund Bergler, the breakdown of relationships was traced to beliefs that middle-aged men and women who had devoted their best years to work and family now deserved to pursue more immediate gratification, to attain ‘happiness in a hurry’—whatever the consequences for those around them. The refrain articulated by Bergler's clients in the 1950s became an established narrative of midlife distress during subsequent decades.

I want happiness, love, approval, admiration, sex, youth. All this is denied me in this stale marriage to an elderly, sickly, complaining, nagging wife. Let's get rid of her, start life all over again with another woman. Sure, I'll provide for my first wife and my children; sure, I'm sorry that the first marriage didn't work out. But self-defence comes first; I just have to save myself.^106^

The midlife crisis—as it emerged in the post-war years—was thus double-edged. Grieving the loss of youthful dreams, middle-aged men and women sought novel pastimes and new partners to reinvigorate stale lives. But, as Bergler and others warned, the outcome was not necessarily greater contentment. Striking out on a fresh course after 40 failed to stem fears of approaching death, leading often to the same set of personal and family challenges from which those at midlife had sought to escape.

## Conclusion

Post-war fiction reveals the stresses and strains experienced by middle-aged men and women as they sought to cope with changes in the life course, conservative reinforcement of the nuclear family, and fluctuating economic opportunities. In David Ely's dystopian novel *Seconds*, published in 1963, Antiochus Wilson pays an organization to change his identity in order to break away from the social and cultural conventions that are suffocating him at home and work.^107^ Disaffected and bored by family and corporate life after returning from World War II, the protagonist in Sloan Wilson's *Man in a gray flannel suit* seeks excitement and comfort elsewhere.^108^ In *The death of Reginald Perrin*, written in 1975 by the English comedy writer David Nobbs, the title character fakes his own death in an attempt to escape from his work and his wife.^109^ Like real midlife crises, fictional ones were not confined to men, however. In novels and short stories such as *The summer before the dark* and ‘To room nineteen’, Doris Lessing explored the parallel pressures experienced by middle-aged women constrained by the tedium of domestic life or by the difficulties of balancing work and family responsibilities in Western patriarchal societies.^110^

Contemporary studies of midlife emphasized the manner in which crises were triggered by awareness of physical and psychological decline and death. Prominent accounts of midlife transitions focused either on the emotional traumas associated with ageing through the second half of life or on the biological changes manifest in middle-aged spread, increased risk of disease and the physiological disruptions associated with the menopause. While such accounts reflected many of the experiences of post-war populations, fears of middle age and the formulation of the midlife crisis should be understood as culturally specific, as products of a particular historical moment in the development of the Western world. Across the middle decades of the twentieth century, standardization of the life cycle generated fresh anxieties about the failure to achieve youthful dreams or to meet socially prescribed milestones in occupational and domestic achievement. At the same time, those at midlife were encouraged to search for personal meaning beyond conventional routes to satisfaction, to shake off the shackles of family and corporate life in order to pursue the American dream of individual happiness and fulfilment. ‘Life begins at 40’ was not merely a catchphrase for those attempting to recapture lost youth. It was also a call to arms for overburdened and disillusioned middle-aged men and women as they sought to recover from the ruins of the war and establish a set of aspirations and values more suited to the political and economic uncertainties of the post-war world.

## Footnotes

1 Elliott Jaques, *The changing culture of a factory* (Tavistock Publications, London, 1951); Elliott Jaques, *Equitable payment: a general theory of work, differential payment, and individual progress* (Heinemann, London, 1961); Wilfred Brown and Elliott Jaques, *Glacier Project papers* (Heinemann, London, 1965).

2 Elliott Jaques, *The life and behavior of living organisms: a general theory* (Praeger, Westport, 2002), p. 3.

3 Douglas Kirsner, ‘The intellectual odyssey of Elliott Jaques from alchemy to science’, *Free Associations* **11**, 179–204 (2004).

4 Elliott Jaques, ‘Death and the midlife crisis’ (1965), reproduced in Elliott Jaques, *Work, creativity, and social justice* (Heinemann, London, 1970), pp. 38–63, at pp. 47–48.

5 *Ibid*., p. 59.

6 *Ibid*. For extensive discussion of the history of the midlife crisis, see Mark Jackson, *The midlife crisis: a history* (Reaktion, London, 2020).

7 See, for example, Pamela Druckerman, ‘How the midlife crisis came to be’, *Atlantic*, 29 May 2018, https://www.theatlantic.com/family/archive/2018/05/the-invention-of-the-midlife-crisis/561203/ (accessed 2 March 2020); Kieran Setiya, *Midlife: a philosophical guide* (Princeton University Press, Princeton, 2017). On the tendency to attribute the term exclusively to Jaques, see Susanne Schmidt, ‘The feminist origins of the midlife crisis’, *Hist. J.* **61**, 503–523 (2018); Susanne Schmidt, ‘The anti-feminist reconstruction of the midlife crisis: popular psychology, journalism and social science in 1970s USA’, *Gender Hist.* **30**, 153–176 (2018). There have been other historical contenders for the role of originator and popularizer of the term. In 1972, a journalist for *New York Magazine* credited the Yale psychologist Daniel Levinson with having ‘christened’ the ‘male midlife crisis’: Linda Wolfe, ‘A time of change’, *New York Magazine*, 5 June 1972, pp. 68–69.

8 Carl Frankenstein, *The roots of the ego: a phenomenology of dynamics and of structure* (Wilkins and Wilkins, Baltimore, 1966), p. 184; Kenneth Soddy, *Men in middle life* (Tavistock Publications, London, 1967), pp. 55–56; Barbara Fried, *The middle-age crisis* (Harper and Row, New York, 1967); Hermann Schreiber, *Midlife crisis. Die Krise in der Mitte des Lebens* (C. Bertelsmann, Munich, 1977); Henry Still, *Surviving the male mid-life crisis* (Thomas Y. Crowell, New York, 1977); Nancy Mayer, *The male mid-life crisis: fresh starts after forty* (Doubleday, New York, 1978); Jim and Sally Conway, *Women in midlife crisis* (Tyndale House, Wheaton, IL, 1983); Edmund Sherman, *Meaning in mid-life transitions* (State University of New York Press, New York, 1984), p. 221. It should be noted that Jaques was not always credited with introducing the term.

9 Henry V. Dicks, *Marital tensions: clinical studies towards a psychological theory of interaction* (Routledge and Kegan Paul, London, 1967), pp. 223–225.

10 Roger L. Gould, ‘The phases of adult life: a study in developmental psychology’, *Am. J. Psychiat.* **129**, 33–43 (1972); Gail Sheehy, *Passages: predictable crises of adult life* (E. P. Dutton, New York, 1974); George E. Vaillant, *Adaptation to life* (Little, Brown, Boston, 1977); Roger L. Gould, *Transformations: growth and change in adult life* (Simon and Schuster, New York, 1978); Daniel J. Levinson, *The seasons of a man's life* (Alfred A. Knopf, New York, 1979).

11 The emotional turbulence of middle age provided the plot-line for many post-war literary accounts of personal, marital and social crises at midlife. See John Updike, *Rabbit, run* (Alfred A. Knopf, New York, 1960); John Updike, *Rabbit redux* (Ballantine Books, New York, 1971); John Updike, *Rabbit is rich* (Ballantine Books, New York, 1981); John Updike, *Rabbit at rest* (Ballantine Books, New York, 1990); Sloan Wilson, *The man in the gray flannel suit* (Simon and Schuster, New York, 1955); Sloan Wilson, *The man in the gray flannel suit II* (Arbor House, New York, 1984); Simone de Beauvoir, *The woman destroyed* (G. P. Putnam's Sons, New York, 1979; first published 1967); David Nobbs, *The death of Reginald Perrin* (Victor Gollanz, London, 1975); *Scenes from a marriage*, dir. Ingmar Bergman, AB Svensk Filmindustri, 1973. For discussion, see Margaret Morganroth Gullette, *Safe at last in the middle years: the invention of the midlife progress novel* (University of California Press, Berkeley, 1988); Julie Levinson, *The American success myth on film* (Palgrave Macmillan, Basingstoke, 2012).

12 G. Stanley Hall, *Senescence: the last half of life* (D. Appleton and Company, New York, 1922); C. G. Jung, *Modern man in search of a soul* (Kegan, Paul, Trench, Trubner and Co., London, 1933); Carl G. Jung, *The integration of the personality* (Kegan, Paul, Trench, Trubner and Co., London, 1940); Erik H. Erikson, *Identity and the life cycle* (W. W. Norton and Company, New York, 1980; first published 1959); Erik H. Erikson, *Childhood and society* (W. W. Norton and Company, New York, 1963; first published 1950).

13 P. B. Medawar, *An unsolved problem of biology* (H. K. Lewis, London, 1952).

14 B. L. Neugarten, ‘Dynamics of transition of middle age to old age: adaptation and the life cycle’, *J. Geriatr. Psychiat.* **4**, 71–100 (1970) at pp. 71–72.

15 *Ibid*., 72.

16 *Ibid*.

17 Tamara K. Hareven, ‘Aging and generational relations: a historical and life course perspective’, *Annu. Rev. Sociol.* **20**, 437–461 (1994). On demographic shifts in the later twentieth century, see Dieter Demey, Ann Berrington, Maria Evandrou and Jane Falkingham, ‘The changing demography of midlife, from the 1980s to the 2000s’, *Popul. Trends* **145**, 1–19 (2011).

18 Michael Anderson, ‘The emergence of the modern life cycle in Britain’, *Soc. Hist.* **10**, 69–87 (1985). On American adulthood, see John Modell, Frank F. Furstenberg, Jr and Theodore Hershberg, ‘Social changes and transitions to adulthood in historical perspective’, *J. Fam. Hist.* **1**, 7–32 (1976).

19 George Carstairs, ‘Lecture 4: the changing role of women’, *This Island Now*, Reith Lectures, 3 December 1961.

20 Richard Titmuss, *Essays on ‘the welfare state’* (George Allen and Unwin, London, 1963; first published 1958), pp. 91–93.

21 Anderson, *op. cit.* (note 18), pp. 69, 73.

22 *Ibid*., p. 76.

23 *Ibid*.

24 Modell, Furstenberg and Hershberg, *op. cit.* (note 18).

25 Howard P. Chudacoff, *How old are you? Age consciousness in American culture* (Princeton University Press, Princeton, 1989); Howard P. Chudacoff, ‘The life course of women: age and age consciousness, 1865–1915’, *J. Fam. Hist.* **5**, 274–292 (1980).

26 Susan J. Matt, *Keeping up with the Joneses: envy in American consumer society, 1890–1930* (University of Pennsylvania Press, Philadelphia, 2003), pp. 1–10.

27 Mass-Observation Archive, ‘Keeping up with the Jones's’, file report 3088, February 1949.

28 Chudacoff (1989), *op. cit.* (note 25), pp. 3–8, 132–137.

29 Steven Mintz and Susan Kellogg, *Domestic revolutions: a social history of American family life* (The Free Press, New York, 1988), pp. 178–179.

30 This is the claim made by Tom Wolfe, ‘The “me” decade and the third great awakening’, *New York Magazine*, 23 August 1976; Ulrich Beck and Elisabeth Beck-Gernsheim, *The normal chaos of love* (trans. Mark Ritter and Jane Wiebel) (Polity Press, Cambridge, 1995); Zygmunt Bauman, *The individualized society* (Polity Press, Cambridge, 2001); Zygmunt Bauman, *Liquid love: on the frailty of human bonds* (Polity Press, Cambridge, 2003).

31 In spite of differences, there were also shared cross-cultural approaches to shaping and measuring the life course: see Charlotte Ikels, Jennie Keith, Jeanette Dickerson-Putman, Patricia Draper, Christine Fry, Anthony Glascock and Henry Harpending, ‘Perceptions of the adult life course: a cross-cultural analysis’, *Ageing Soc.* **12**, 49–84 (1992).

32 Tamara K. Hareven, ‘Family time and historical time’, *Daedalus* **106**, 57–70 (1977), at p. 58.

33 George Orwell, ‘The art of Donald McGill’, *Horizon*, September 1941, pp. 153–163. On class differences in ageing across time, see Pat Thane, ‘Social histories of old age and aging’, *J. Soc. Hist.* **37**, 93–111 (2003).

34 Orwell, *op. cit.* (note 33).

35 Kiyomo Morioka, ‘Life cycle patterns in Japan, China, and the United States’, *J. Marriage Fam.* **29**, 595–606 (1967). On divorce in Japan, see Harald Fuess, *Divorce in Japan: family, gender and the state, 1600–2000* (Stanford University Press, Stanford, 2004).

36 Morioka, *op. cit.* (note 35).

37 Mintz and Kellogg, *op. cit.* (note 29), pp. 141–144.

38 Steven Mintz, *The prime of life: a history of modern adulthood* (Belknap Press, Cambridge, MA, 2015), pp. 135–140.

39 Thomas S. Weisner and Lucinda P. Bernheimer, ‘Children of the 1960s at midlife: generational identity and the family adaptive project’, in *Welcome to middle age!* (ed. Richard A. Schweder), pp. 211–257 (University of Chicago Press, 1998), at p. 216.

40 Thomas R. Cole, *The journey of life: a cultural history of aging in America* (Cambridge University Press, 1992).

41 G. Stanley Hall, ‘The dangerous age’, *Pedagog. Semin.* **28**, 275–294 (1921), at p. 275.

42 Margaret Morganroth Gullette, *Aged by culture* (University of Chicago Press, 2004); Margaret Morganroth Gullette, *Declining to decline: cultural combat and the politics of the midlife* (University Press of Virginia, Charlottesville, 1997); and Margaret Morganroth Gullette, *Ending ageism, or how not to shoot old people* (Rutgers University Press, New Brunswick, 2018).

43 Gullette, *op. cit.* (note 11), pp. 59–84.

44 Updike, *Rabbit is rich, op. cit.* (note 11), p. 208.

45 For examples of women's fictional writings about midlife in the post-war decades, see de Beauvoir, *op. cit.* (note 11); Doris Lessing, *The summer before the dark* (Jonathan Cape, London, 1973).

46 Levinson, *op. cit.* (note 10); Daniel J. Levinson, *The seasons of a woman's life* (Ballantine Books, New York, 1996). See also Hilda L. Smith, ‘“Age”: a problematic concept for women’, *J. Women's Hist.* **12**, 77–86 (2001).

47 Julia Kristeva, ‘Women's time’, *Signs: J. Women Cult. Soc.* **7**, 13–35 (1981).

48 Ikels et al., *op. cit.* (note 31), pp. 61–62.

49 Lynne Segal, ‘Temporal vertigo: the paradoxes of ageing’, *Stud. Gend. Sex.* **15**, 214–222 (2014).

50 *Ibid*., pp. 218–219, citing Greer.

51 *Ibid*., pp. 219–220. Segal was referring especially to autobiographical works by Simone de Beauvoir and Doris Lessing.

52 Alice S. Rossi, ‘Life-span theories and women's lives’, *Signs: J. Women Cult. Soc.* **6**, 4–32 (1980), at pp. 5–6.

53 Marjorie Fiske Lowenthal, Majda Thurnher and David Chiriboga (eds), *Four stages of life: a comparative study of women and men facing transitions* (Jossey-Bass, San Francisco, 1976), p. x.

54 Rossi, *op. cit.* (note 52), p. 7.

55 Charlotte Bühler, ‘The human course of life in its goal aspects’, *J. Humanist. Psychol.* **4**, 1–18 (1964).

56 *Ibid*., p. 1.

57 John Benson, *Prime time: a history of the middle aged in twentieth-century Britain* (Addison Wesley Longman, Harlow, 1997), pp. 32–36.

58 Thomas C. Desmond, ‘America's unknown middle-agers’, *New York Times*, 29 July 1956, pp. 5, 42–43.

59 Clark Tibbitts, ‘Life begins at 50’, *Buffalo Courier Express*, 15 Mary 1960.

60 ‘The pleasures and perils of middle age’, *Time*, 29 July 1966, http://time.com/3105861/lauren-bacall-middle-age/ (accessed 30 January 2019).

61 Nixola Greeley-Smith, ‘Now is the time for all women to train for the duties that war time may bring’, *Pittsburgh Press*, 10 April 1917, p. 20.

62 Mrs Theodore Parsons, *Brain culture through scientific body building* (American School of Mental and Physical Development, Chicago, 1912); Mrs Theodore Parsons, *Making the body think* (Kelmscott Press, New York, 1926).

63 Greeley-Smith, *op. cit.* (note 61).

64 Forbes Lindsay, ‘The man of fifty’, *Harper's Weekly*, 53, 16 October 1909, pp. 15–16.

65 See James Stark, *The cult of youth: anti-ageing in modern Britain* (Cambridge University Press, 2019); Aimee Medeiros and Elizabeth Siegel Watkins, ‘Live longer better: the historical roots of human growth hormone as anti-aging medicine’, *J. Hist. Med. Allied Sci.* **73**, 333–359 (2018). Middle-aged spread became a popular term in the 1930s: Benson, *op. cit.* (note 57), pp. 17–19.

66 ‘So often … life begins at forty’, *Tatler*, 27 June 1934, p. 605.

67 ‘Forty-phobia (fear of the forties)’, *Times*, 28 April 1938, p. 19; ‘Fortyphobia’, *Maclean's*, 1 April 1938, http://archive.macleans.ca/article/1938/4/1/fortyphobia (accessed 18 February 2019).

68 On concerns about body weight and heart disease in working men, see Jane Hand, ‘Marketing health education: advertising margarine and visualising health in Britain from 1964–c.2000’, *Contemp. Br. Hist.* **31**, 477–500 (2017); Jane Hand, ‘“Look after yourself”: visualising obesity as a public health concern in 1970s and 1980s Britain’, in *Balancing the self: medicine, politics and the regulation of health in the twentieth century* (ed. Mark Jackson and Martin Moore), pp. 95–124 (Manchester University Press, 2020).

69 Susan Sontag, ‘The double standard of aging’, *Saturday Review*, 23 September 1972, pp. 29–38, at p. 35.

70 *Ibid*., 31.

71 Walter B. Pitkin, *Life begins at forty* (McGraw-Hill, New York, 1932).

72 James Truslow Adams, *The epic of America* (Little, Brown, and Company, Boston, 1931). See also Sarah Churchwell, *Behold, America: a history of America First and the American dream* (Bloosmbury, London, 2018).

73 Adams, *op. cit.* (note 72), p. 415.

74 *Ibid*., pp. 416–417.

75 James Truslow Adams, *Our business civilization: some aspects of American culture* (Albert and Charles Boni, New York, 1929), pp. 289–306, at p. 299.

76 *Life begins at 40*, dir. George Marshall, USA, Fox Film Corporation, 1935, available on the Internet Movie Database, https://www.imdb.com/title/tt0026629/ (accessed 5 March 2019).

77 *Life begins at eight-thirty*, dir. Irving Pichel, USA, 20th Century Fox, 1942, available on the Internet Movie Database, https://www.imdb.com/title/tt0034976/ (accessed 1 February 2019).

78 Alva Myrdal and Viola Klein, *Women's two roles: home and work* (Routledge and Kegan Paul, London, 1968; first published 1956), p. 17.

79 Sinclair Lewis, *Babbitt* (Bernhard Tauchnitz, 1922); Babbitt's moment of enlightenment appears on p. 288. The term ‘Babbittry’ became popular in America as a descriptor of narrow-minded materialism.

80 Pitkin, *op. cit.* (note 71), p. 7.

81 *Ibid*., p. 24.

82 Mark Abrams, ‘British standards of living’, *Current Affairs*, 63, 18 September 1948; Denys Thompson, ‘The importance of leisure’, *Current Affairs*, 72, 22 January 1949; Mass-Observation, ‘A report of work and leisure’, file report 3067, November 1948.

83 Pitkin, *op. cit.* (note 71), p. 107.

84 *Ibid*.

85 *Ibid*., p. 86.

86 *Ibid*., p. 149.

87 *Ibid*., pp. 49, 107.

88 Walter Pitkin Jr, *Life begins at fifty* (Simon and Schuster, New York, 1965), pp. 21–22, emphasis in original.

89 Sue Currell, ‘Depression and recovery: self-help and America in the 1930s’, in *Historicizing lifestyle: mediating taste, consumption and identity from the 1900s to 1970s* (ed. David Bell and Joanne Hollows), pp. 131–144 (Ashgate, Aldershot, 2006).

90 See, for example, Grace Loucks Elliott, *Women after forty: the meaning of the last half of life* (Holt, New York, 1936); Boris Sokoloff, *Middle age is what you make it* (Greystone Press, New York, 1938); Gayelord Hauser, *Look younger, live longer* (Faber and Faber, London, 1950); Maxine Davis, *Get the most out of your best years* (Dial Press, New York, 1960); Nancy Phelan and Michael Volin, *Yoga over forty* (Harper & Row, New York, 1965); Desmond Dunne, *Yoga for everyman: how to have long life and happiness* (Gerald Duckworth, London, 1951).

91 Neil Brierley, *Relaxation for men: tension in modern living* (Ward Lock, London, 1965), p. 7.

92 E. J. Boome and M. A. Richardson, *Relaxation in everyday life* (Methuen, London, 1938), pp. 1, 99.

93 Phelan and Volin, *op. cit.* (note 90), p. 48.

94 Hauser, *op. cit.* (note 90), pp. 188–197.

95 Nadina R. Kavinoky, ‘A balanced life for mental health’, *Marriage Fam. Living* **6**, 41–42, 58, 64 (1944).

96 *Ibid*.

97 Leslie J. Tizard and Harry J. S. Guntrip, *Middle age* (George Allen and Unwin, London, 1959), pp. 60–61.

98 See the discussion in Tom Lutz, ‘“Sweat or die”: the hedonization of the work ethic in the 1920s’, *Am. Lit. Hist.* **8**, 259–283 (1996).

99 Frederick Cooper, ‘Medical feminism, working mothers, and the limits of home: finding a balance between self-care and other-care in cross-cultural debates about health and lifestyle, 1952–1956’, *Palgrave Commun.* **2** (2016), http://www.nature.com/articles/palcomms201642 (accessed 8 August 2017).

100 Pitkin, *op. cit.* (note 71), p. 112.

101 *Ibid*., p. 115. Pitkin's son also recognized the specific problems of middle-aged women: Pitkin Jr, *op. cit.* (note 88), pp. 16, 211–213.

102 Pitkin, *op. cit.* (note 71), p. 112.

103 Marion Hilliard, *Women and fatigue* (Pan Books, London, 1963; first published 1958), p. 82. On Hilliard, see Kaitlynn Mendes, ‘Reading *Chatelaine*: Dr. Marion Hilliard and 1950s women's health advice’, *Can. J. Commun.* **35**, 515–531 (2010).

104 Marion Hilliard, ‘Stop being just a housewife’, *Chatelaine*, September 1956, pp. 11, 90–95.

105 William Brown, *Psychological methods of healing: an introduction to psychotherapy* (University of London Press, London, 1938), pp. 158–160; J. H. Wallis, *Someone to turn to* (Routledge and Kegan Paul, London, 1961), p. 100.

106 Edmund Bergler, *The revolt of the middle-aged man* (Bernard Hanison, London, 1958), pp. 75–76.

107 David Ely, *Seconds* (Harper Voyager, New York, 1963).

108 Wilson (1955), *op. cit.* (note 11).

109 Nobbs, *op. cit.* (note 11).

110 Lessing, *op. cit.* (note 45); Doris Lessing, ‘To room nineteen’, in Doris Lessing, *To room nineteen: collected stories volume one* (Flamingo, London, 1994), pp. 352–386.

